# What evidence exists for the impact of climate change on the physiology and behaviour of important aquaculture marine crustacean species in Asia? A systematic map protocol

**DOI:** 10.1186/s13750-022-00263-1

**Published:** 2022-03-12

**Authors:** Mohamad Nor Azra, Mohd Iqbal Mohd Noor, Jacqualyn Eales, Yeong Yik Sung, Mazlan Abd Ghaffar

**Affiliations:** 1 Institute of Marine Biotechnology (IMB), Universiti Malaysia Terengganu (UMT), 21030 Terengganu, Malaysia; 2 Climate Change Adaptation Laboratory, Institute of Marine Biotechnology (IMB), Universiti Malaysia Terengganu (UMT), 21030 Terengganu, Malaysia; 3 Faculty of Business Management, Universiti Teknologi MARA (UiTM) (Pahang), 27600 Raub, Pahang Malaysia; 4 Institute for Biodiversity and Sustainable Development, Universiti Teknologi MARA (UiTM), 40450 Shah Alam, Selangor Malaysia; 5 European Centre for Environment and Human Health, University of Exeter Medical School, Knowledge Spa, Royal Cornwall Hospital, Truro, TR1 3HD UK; 6 Faculty of Science and Marine Environment, Universiti Malaysia Terengganu (UMT), 21030 Terengganu, Malaysia; 7 Higher Institution Centre of Excellence (HICoE), Institute of Tropical Aquaculture and Fisheries (AKUATROP), Universiti Malaysia Terengganu (UMT), 21030 Terengganu, Malaysia

**Keywords:** Aquatic, Arthropod, Environmental sciences, Global warming, Invertebrate, Marine biodiversity, Metabolism, Movement, Predatory, Temperature

## Abstract

**Background:**

Climate is one of the most important driving factors of future changes in terrestrial, coastal, and marine ecosystems. Any changes in these environments can significantly influence physiological and behavioural responses in aquatic animals, such as crustacea. Crustacea play an integral role as subsistence predators, prey, or debris feeders in complex food chains, and are often referred to as good indicators of polluted or stressed conditions. They also frequently have high production, consumption, and commercial significance. However, crustacean’s responses to climate change are likely to vary by species, life-history stage, reproduction status and geographical distribution. This map is undertaken as part of the Long-Term Research Grant project which aims to identify any interactive effect on physiological compensation and behavioural strategy of how marine organisms, especially crustaceans, deal with stress from environmental change. Our proposed map will aim to outline the evidence currently existing for the impacts of climate change on the physiology and behaviour of important aquaculture crustacean species within Asia.

**Methods:**

We will document peer-reviewed articles in English using published journal articles and grey literature. Two bibliographic databases (Scopus and Web of Science) and multiple organizational websites with Google scholars will be searched. The systematic map protocol will follow in accordance with the Collaboration for Environmental Evidence Guidelines and Standards. Literature will be screened at the title, abstract, and full-text level using pre-defined inclusion criteria. The map will highlight marine crustacea physiological compensation and behavioural strategies to cope with climate change. It will also improve our knowledge of the available evidence and current gaps for future research recommendations.

**Supplementary Information:**

The online version contains supplementary material available at 10.1186/s13750-022-00263-1.

## Background

The Food and Agriculture Organization (FAO) of the United Nations (UN) defines climate change as “A change in the state of the climate that can be identified by changes in the mean or the variability of its properties, and that persists for an extended period, typically decades or longer”. Another definition of climate change may refer “to any change in climate over time, whether due to natural variability or as a result of human activity”, according to the United Nations Framework Convention on Climate Change (UNFCCC). The upcoming IPCC (Intergovernmental Panel in Climate Change) Sixth Assessment Report, currently due for release in 2022, is expected to encourage numerous climate change-related studies. In addition, the United Nations (UN) General Assembly has introduced 17 UN SDG (Sustainable Development Goals) to all governments and private organizations. One of the important SDGs related to climate change is SDG 13, Climate Action, with the official aim to “Take urgent action to combat climate change and its impacts”. Thus, studies that review and synthesize evidence for climate change impacts are sorely needed to understand the current body of knowledge surrounding climate change. We intend to fulfill that goal here.

Climate is one of the most important factors driving future change in terrestrial, coastal, and marine ecosystems, affecting the ecosystem itself and the resources they provide [1–4]. Coastal and marine environments are highly valuable economically, with an estimated annual global value of $20.9 trillion USD [5, 6]. Notwithstanding impacts on ecosystem services, climate changes can affect marine ecosystem production, especially in countries and societies that depend on resources from marine fisheries [7]. Fisheries focused on arthropods may be particularly sensitive, as arthropods tend to rely on water quality stability [8, 9]. Therefore, changes in marine environments could significantly influence an animal’s physiological and behavioural responses [10, 11], including crustaceans (e.g. [12, 13]). There is growing evidence showing that marine crustacean species may be especially sensitive to climate changes, such as climate warming, because they have narrower thermal niches and are currently living closer to their thermal maximum capacity [8, 14–17].

Crustaceans are the most species rich group of marine animals, living from the deepest ocean depths to muddy wetlands [18]. Indeed, there are an estimated 150,000 species worldwide, with only 42,000 species described thus far [19]. Crustaceans play an important role as predators, prey, or debris feeders in complex food chains and are often referred to as (i) global invasive potentials, (ii) good indicators of polluted or stressed conditions, and (iii) frequently have high production, consumption, and commercial significance [20–22]. Because of their broad distribution around coastal, estuarine, and intertidal habitats, crustaceans are strongly affected by climate-induced environmental changes, such as temperature rise, sea level rise, heatwaves, sea surface temperature, etc. Understanding the direct effects of climate change and variability on crustaceans is an important consideration for effective aquaculture and fisheries management and conservation. Climate change can lead to significant changes in maximum catch potential in the world’s exclusive marine economic zones [23], thus directly affecting fisheries-related activities in the future.

**Fig. 1:**
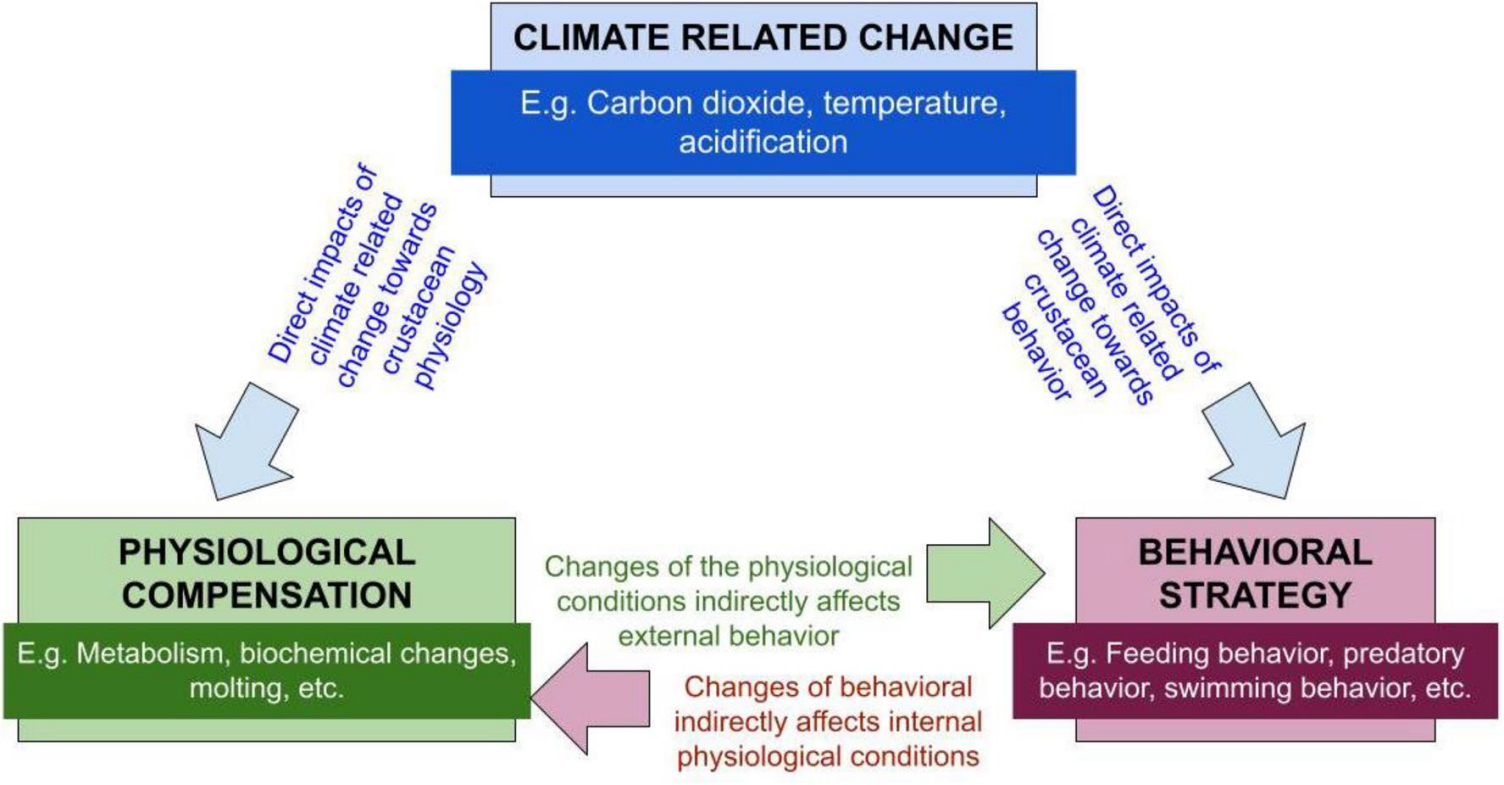
Conceptual framework representing direct impacts (blue arrows) and relationships between (magenta and green arrows) climate change and crustacean’s physiological and behavioral responses

Climate warming poses a growing threat to marine crustacean populations by intensifying habitat loss and interfering with feeding, molting, reproductive performance, biochemical compositions, locomotor behavior and survival [24–28]. Understanding crustaceans’ ability to cope with climate-related change is important to predict the likelihood of range shifts and extinction rates owing to environmental stress. Acclimation, a plastic response, is one-way organisms cope with adverse environmental conditions through the modification of behavioural, physiological, biochemical, and fitness characteristics in relation to environmental change [29]. Under current climate projections, climate impact is unavoidable and physiological adaptability or changes in behaviour will be key for many organisms to survive. Thus, understanding the physiological and behavioural adaptations required of marine organisms is important to ensure their continued conservation and wellbeing. However, crustacean responses to climate variability and change are not uniform and vary by species, life-history stage, reproduction status, and geographical distribution. Here, we generate foundational knowledge with a systematic map to describe what is already known and identify important research gaps regarding marine crustacean physiological and behavioural adaptability. We provide insights on the various responses of crustaceans to climate change and likely future trajectories in species vulnerability, abundance and composition. Systematic maps are increasingly being used to describe the abundance and distribution of primary literature [5, 30] in various fields [4, 31], regionally and globally. Figure 1 shows the conceptual framework for our study, representing direct impacts of climate change on crustacean’s physiological and behavioral conditions.

### Stakeholder involvement

**Fig. 2:**
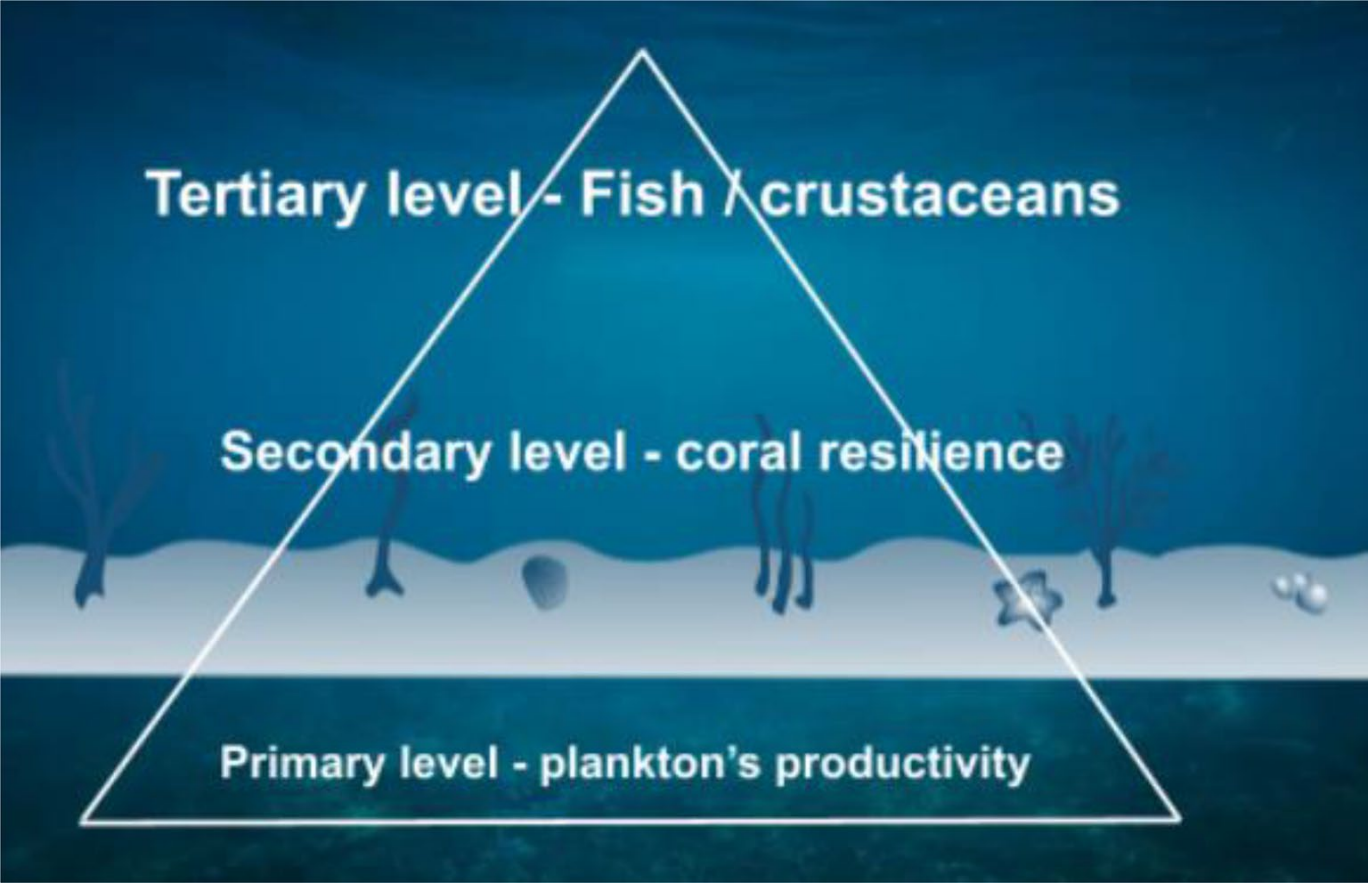
Example of shallow marine ecosystems, where plankton productivity forms the primary level, coral resilience is the secondary level, and fish as well as crustaceans (or other ecologically important fauna) make up the tertiary level

This systematic map is part of the research program “Ocean Climate Change Potential Risk, Impact and Adaptation Towards Marine and Coastal Ecosystem Services in Malaysia”. This is a multi-organizational team project, involving representatives from Universiti Malaysia Terengganu (UMT), Universiti Malaya (UM), Universiti Sains Malaysia (USM), and International Islamic University Malaysia (IIUM). Universiti Malaysia Terengganu (UMT) is responsible for leading the mapping process. The authors of this systematic map and its protocol include members and senior representatives from each of the groups mentioned above and international academicians. The main authors discussed and refined the scope of the map during several meetings at the initial planning stage of the project. This project is financed by the Ministry of Higher Education Malaysia under the research program of Long-term Research Grant Scheme, 2020–2024. The project’s aim is to chart the impact of climate change and related impacts, including but not limited to acidification, temperature changes, and salinity changes. We focus on how climate change affects the physiological and behavioral responses of organisms living in shallow marine ecosystems,’ starting with plankton’s productivity as a primary level, coral resilience as a secondary level, and fish and other ecologically important marine species (e.g. crustaceans) as a tertiary level (Fig. 2). Our systematic map will help policy makers improve aquaculture and fisheries management by understanding marine organism’s adaptability under environmental change.

### Objective of the review

We aim to identify, catalogue, and describe the existing evidence for climate impacts on marine crustacean species important to aquaculture in Asia, specifically focusing on physiological and behavioural compensation strategies. Our map will outline the factors that might influence animal physiology and will highlight areas where there is little existing research. Ultimately, this systematic map will provide direction for scientific, philanthropic, government, and non-government organizations on how to invest limited resources in programs, policies, and research towards the sustainable management of crustaceans in marine ecosystems.

We seek to answer the following review question: “*What evidence exists for the impact of climate change on the physiology and behaviour of important aquaculture marine crustacean species in Asia?*”. The components of our primary research question are broken down in Table 1.

**Table 1:** Components of primary research questions

Population	Exposure	Outcome
Commercially important aquaculture’s marine crustaceans’ species in Asia	Climate change	Physiological compensation; behavioral strategy

## Methods

Our systematic map protocol has been developed in accordance with the Guideline and Standards for Evidence Synthesis in Environmental Management, latest version of 5.0 [32], and conforms to ROSES reporting standards [33] (see Additional file 1).

### Bibliographic databases

The literature search for this systematic map will be undertaken in two databases: web of Science and Scopus. We will use the Science Citation Index Expanded (SCI-EXPANDED), part of the Web of Science Core Collection, and will run searches based on the “topic” (TS) field. This includes article titles, abstracts, keywords, and “KeyWords Plus” (automatically generated terms pulled from the titles of cited articles). In Scopus, article titles, abstracts, and keywords will be searched using the search string outlined below, and all years of data will be included.

The three (3) sub-strings (Population, Exposure and Outcome) of the search equation will be combined with “AND” and searched in title, abstract and keywords of the Scopus and WOS databases using the search tags “TITLE-ABS-KEY” and “TS”, respectively.

### Web-based search engines

Additional searches will be performed using Google Scholar, with the aid of the software Publish and Perish to retrieve scientific and grey literature. We adapted the search string to correspond to what the review team deemed as the most important keywords (“marine crustacean” AND “climate change” AND “behavioral” AND “psychological” AND “asia”) and searched in the section “keywords” which searches in the title, abstract, and body text. We will export the first 300 search hits, in line with recommendations by Haddaway et al*.* [33].

### Specialist websites

The following specialist organizations will be searched for relevant publications.

1. https://www.fao.org/climate-change/en/.
2. https://www.iucn.org/resources/publications.
3. https://unfccc.int/documents.
4. https://www.ipcc.ch/documentation/.
5. http://www.thegef.org/.
6. https://www.un.org/climatechange.
7. https://www.iisd.org/focus-areas/climate.
8. https://www.unsdsn.org/.
9. https://www.ipbes.net/.
10. https://climate-adapt.eea.europa.eu/.
11. https://public.wmo.int/en.
12. https://oceanfdn.org/ocean-and-climate-change/.
13. https://www.climate.gov/.
14. https://oceanclimatechange.umt.edu.my/.
15. https://www.mbari.org/climate-change/.
16. https://www.usgs.gov/.
17. https://www.carbonbrief.org/.
18. https://www.climatecentral.org/.
19. https://www.worldwildlife.org/threats/effects-of-climate-change.
20. https://ocean-climate.org/en/home-2/.
21. https://www.imf.org/en/home.
22. https://www.greenclimate.fund/.
23. https://www.climatecentre.org/resources/publications/.
24. https://climate.copernicus.eu/.

A call for grey literature will be conducted through the Scientific Council of our project to find non-peer-reviewed literature, articles from professional journals of climate change. All members of the Scientific Council have been asked to provide documents that they considered relevant to the subject.

### Searching for articles and comprehensiveness of the searches

The final search string that produced the highest efficiency was built using the sub-string shown below and returned 1845 hits as of 25th January 2022 (Additional file 2). A scoping exercise was conducted in the Web of Science Core Collection database to build-up the search strings. A benchmark list of 20 articles of known relevance to systematic map objectives was screened against scoping search results to examine the search string capability in locating relevant articles (Additional file 3) [34]. Improvement of search terms is done based on the scoping exercise to make sure no relevant articles will be missed during actual mapping search. All 20 articles (100% comprehensiveness) were retrieved with the search term at Web of Science Database. Improvement of search terms will be done based on the scoping exercise to make sure no relevant articles will be missed during actual mapping search.

Based on the scoping exercise at Additional File 2, the primary objective of the map focuses on climate change’s impact on the physiology and behavior of commercially important aquaculture’s marine crustaceans’ species in Asia, only the Population and Exposure terms were included in the search string. No time or document type restriction will be applied. The search term string is presented below.

### Search terms

#### Population terms

##### Selection of marine crustacean species in Asia

Selection of the marine crustacean species were based on the Food and Agriculture Organization (FAO) catalogue published in FAO Cultured Aquatic Species Information Programme (CASIP). According to this dataset, there are about seven (7) cultured crustacean species in Asia that live in the marine environment or have part of their life cycle in marine habitats (i.e. brackish water). The following are the included search terms relating to species identity:

TS = ((“Chinese river crab”) OR (“Chinese mitten crab”) OR (“Shanghai hairy crab”) OR (“Eriocheir sinensis”) OR (“Giant river prawn”) OR (“Giant freshwater prawn”) OR (“Macrobrachium rosenbergii”) OR (“giant tiger prawn”) OR (“Asian tiger shrimp”) OR (“black tiger shrimp”) OR (“black tiger prawn”) OR (“Penaeus monodon”) OR (“Indian white prawn”) OR (“Indian prawn”) OR (“Penaeus indicus”) OR (“Giant mud crab”) OR (“mud crab”) OR (“mangrove crab”) OR (“black crab”) OR (“Indo-Pacific swamp crab”) OR (“Scylla serrata”) OR (“Scalloped spiny lobster”) OR (“spiny lobster”) OR (“furry lobsters”) OR (“Panulirus homarus”) OR (“Whiteleg shrimp”) OR (“Pacific white shrimp”) OR (“King prawn”) OR (“Litopenaeus vannamei”) OR (“Penaeus vannamei”)).

#### Exposure terms

##### Selection of exposure terms related to climate change issue

We focused on three main climate change issues: increasing atmospheric carbon dioxide, rising or changing temperatures, and ocean acidification. Accordingly, our search terms included:

TS = ((“carbon dioxide”) OR (“temperature”) OR (“acidification”)).

#### Article screening and study eligibility criteria

##### Screening process

Article’s screening will be done in two stages: (i) title and abstract level and (ii) full text level. At each level, a study will be assessed using eligibility criteria listed below. The first level involves a review of an article’s title and abstract. Studies that meet the inclusion criteria will be reviewed at the second stage (full text level). Any articles that seem to be unclear on its criteria and relevancy will be included and reviewed at the full text stages. A record of inclusion/exclusion will be taken for each stage. Articles that are excluded in the full-text stage will be recorded with the reasons for exclusion as an additional file. This two-stage screening will be conducted by two reviewers.

To ensure consistency and accuracy of inclusion/exclusion decisions throughout the screening process, a Kappa test will be performed. A random set of 100 articles (or 10% of the total, whichever is the greater) will be selected and screened by each of the reviewers independently at both screening stage. Consistency in decisions will be analyzed using the Cohen’s Kappa (K) test [35]. The result is deemed as ‘substantial’ if K is between 0.61 and 0.80) or ‘almost perfect’ agreement if K is between 0.81 and 1.0. To ensure the best possible outcome, disagreements will be discussed and resolved between reviewers before beginning the screening process regardless of the Kappa value results. In the case of retrieved publication that was authored or co-authored by one or more members of the review team, the publication will be referred to another reviewer for assessment. The team will also use proper consideration when interpreting Kappa scores due to the limited ability of the test to appropriately estimate consistency in agreement, particularly when proportion of inclusion is low.

### Eligibility criteria

Articles meeting the following criteria will be included in the final systematic map:

#### Eligible populations

The systematic map only includes studies focused on seven species (*Eriocheir sinensis, Macrobrachium rosenbergii, Penaeus monodon, Penaeus indicus, Scylla serrata, Panulirus homarus, Litopenaeus vannamei or Penaeus vannamei*) that are commercially important aquaculture’s marine crustaceans’ species in Asia as the subject of the research. No other marine organisms will be considered. If a study includes results for both a crustacean and a non-crustacean, this will not be included.

#### Eligible exposures

This systematic map only includes studies that focus on direct climate variables and their derivatives. This refers to the direct impact of carbon dioxide, temperature, or acidification on crustacean species. Articles that explore the effects of changes in secondary variables without linking those changes to direct variables will also be excluded.

#### Eligible comparators

No comparator is required for this map.

#### Eligible outcomes

We will only include studies that focus on crustacean behavior and physiology. Studies will be accepted if they report measures of physiological response and behavioral response, which could be represented by statistical demography data. Those studies reporting only the effects on other outcomes will be excluded. Where outcomes are only modeled or predicted, they are not eligible.

#### Eligible study designs

We will include experimental, quasi-experimental, non-experimental, narrative, and observational studies. Due to our team’s resource capability and the plethora of potential information related to climate change, we will exclude laboratory experiments.

### Study validity assessment

The critical appraisal of study quality and strength will not be carried out formally for this systematic map. However, the information regarding study design elements will be coded. The purpose of this systematic map is to do a thorough narrative synthesis and to inform future systematic reviews.

### Data coding strategy

Meta-data extraction and coding will be done for all studies that pass the inclusion/exclusion criteria in the full-text screening stage. As before, meta-data extraction and coding will be performed by two members of the team. At least 10% of articles will be double screened and checked for consistency. Any discrepancies will be discussed and reviewers will continue once data extraction forms have been clarified, and reviewers are interpreting the forms consistently. Concerning missing data, if data is not sufficiently detailed or simply unreported, reviewers will attempt to contact primary authors to obtain missing data. Finally, the following meta-data will be extracted for each paper:

Bibliographic information:

- Study ID (unique numeric ID assigned to each article).
- Coder ID (unique ID assigned to each reviewer).
- Citation information
- Study content (study, review, meta-analysis).

### Study design

- Study objective
- Study timeframe
  – Study starts and end dates
  – Study duration (before and after data collection)
- Population information
  – Study location (country, state/province, site name and location, habitat type, climate zone)
  – Crustacean species
  – Demographic information
  – Field investigation database
  – Site comparison
- Exposure variables
  – Direct climate variables (carbon dioxide, temperature, acidification)
  – Direct climate variable derivatives
- Outcome components and subcomponents measured
  – Behavioral performances
  – Physiological performances

### Study mapping and presentation

The final systematic map will explain the methods and database, and provide summary figures and tables of the study characteristics for all evidence identified. The map will particularly highlight the direct effects of climate change issues on crustacean’s physiological compensation and behavioural strategy. Our map will improve our knowledge of the available evidence and current gaps for future research recommendations. Based on our results, recommendations will be made on how to prioritize future research to mitigate climate change impact. The full database containing the information extracted from each study will also be made available for download.

## Supplementary Information

**Additional file 1: **ROSES form.

**Additional file 2: **Evolution of search string.

**Additional file 3: **Article for benchmarking.

## Data Availability

Not applicable.
