# Supplementary material for: What evidence exists for the impact of climate change on the physiology and behaviour of important aquaculture marine crustacean species in Asia? A systematic map protocol
Source: Environ Evid. 2022 Mar 12;11:9. doi: 10.1186/s13750-022-00263-1 (PMC11378817; doi:10.1186/s13750-022-00263-1)
Supplement: Supplementary file 2 — Additional file 2: Evolution of search string. [file 13750_2022_263_MOESM2_ESM.docx]

**Additional File 2: Evolution of search string.**

**What evidence exists for the impact of climate change on the physiology and behaviour of important aquaculture marine crustacean species in Asia? A systematic map protocol**

**Additional File**

This document summarizes the main steps taken to identify search terms and develop the search string through scoping exercises.

**Scoping**

An extensive set of keywords was compiled using expert knowledge, relevant journal articles and books and by consulting with the stakeholder group members. The keywords were combined to develop a search string relevant to the following predefined elements generated by the primary question:

Population: Asia Crustacean’s species in marine environment

Intervention: Climate Change

Outcome: studies focusing on crustacean’s physiological compensation and behavioral strategy

A scoping exercise was conducted using Web of Science Core Collections to modify the search terms by examining for specificity and sensitivity of alternate terms, wildcards and Boolean operators. The asterisk was used as a wildcard to allow for singular or plural words to be identified in the same search. A list of alternative terms was established with the aid of a thesaurus and screening of relevant citations.

Various iterations of the search string were tested against a ‘benchmark’ list of 20 key articles selected to represent a range of academic studies focus on climate change and crustacean’s species, covering a range of authors, journals, and research topics relevant to the scope of the question. Each test search was carefully recorded as a percentage of the test list and saved for access later. The search was finalized once all the studies in the ‘benchmark’ list were found.

The final search string was composed of terms related to the ‘Population’, ‘Intervention’ and ‘Outcome’ elements. The basic terms relating to the ‘Population’ elements of asia marine crustacean were too broad and resulted in a large number of irrelevant articles. The extensive list of keywords was reduced significantly to a shorter list of key terms that encompassed most crustacean’s ecology marine interventions. The list was reduced by including and removing individual keywords to determine the impact on the final search results. Those keywords that resulted in only a few extra articles were not included, and those that resulted in at least 50 more articles were included. The search term for crustacean also includes the Food and Agriculture Organization of the United Nations keyword for crustaceans’ taxonomic group to capture the majority of crustaceans research articles.

To deal with specific exposure phrase, this article uses three way of search strings ranging from restrictive to broadest approach. The three-specific way of search are: (i) using quotes (e.g. “climate change”) ; (ii) using the OR operator and (iii) using the AND operator (e.g. (climate AND behavioral)). Before accepting the final search string, an exercise was conducted to review the potentially relevant articles that are excluded when using the single term “crustaceans”. The titles and abstracts of the first 100 articles in the search result were reviewed briefly to identify relevant articles. Only 5% of the articles were deemed moderately relevant to the study, therefore the final search string was approved. Additionally, the search will be limited to articles published from 2010 onwards in order to focus the study on the last decade which has seen a noticeable increase in research that integrates climate changes datasets to better understand focus of study.

| **Step** | **PECO** | **Search type** | **Search string** | **Number of publications retrieved in WOS CC** | **Comprehensiveness**  **(Based on 20 articles indexed in WOS CC) *** | **Comments** |
| --- | --- | --- | --- | --- | --- | --- |
| **1** | Population | TS | (crustacea*) | 56,504 | 9/20  (45%) | High volume and key word use only refer to crustaceans hence the low comprehensiveness. |
|  |  | TS | ((“Chinese river crab”) OR (“Chinese mitten crab”) OR (“Shanghai hairy crab”) OR (“Eriocheir sinensis”) OR (“Giant river prawn”) OR (“Giant freshwater prawn”) OR (“Macrobrachium rosenbergii”) OR (“giant tiger prawn”) OR (“Asian tiger shrimp”) OR (“black tiger shrimp”) OR (“black tiger prawn”) OR (“Penaeus monodon”) OR (“Indian white prawn”) OR (“Indian prawn”) OR (“Penaeus indicus”) OR (“Giant mud crab”) OR (“mud crab”) OR (“mangrove crab”) OR (“black crab”) OR (“Indo-Pacific swamp crab”) OR (“Scylla serrata”) OR (“Scalloped spiny lobster”) OR (“spiny lobster”) OR (“furry lobsters”) OR (“Panulirus homarus”) OR (“Whiteleg shrimp”) OR (“Pacific white shrimp”) OR (“King prawn”) OR (“Litopenaeus vannamei”) OR (“Penaeus vannamei”)) | 19,999 | 20/20  (100%) | High volume and high comprehensiveness. Need to reduce volume to make the map exercise more manageable |
| 2 | *Population + Exposure* | TS | **POPULATION AND = ((“carbon dioxide”) OR (“temperature”) OR (“acidification”))** | 1845 | 20/20  (100%) | To reduce the search hits. We added a context. Even though the comprehensiveness is maximum, articles volume not yet acceptable. The author decides to add one more outcome block in order to mitigate this issues. |
| 3 | Population + *Exposure* + Outcome | TS | **POPULATION AND EXPOSURE AND =** ((behavio*) OR (swim*) OR (recover*) OR (predat*) OR (feed*) OR (move*) OR (forag*) OR (“locomot*”) OR (“motor* activit*”) OR (mat*) OR (court*) OR (aggress*) OR (burrow*) OR (strength*) OR (fight*) OR (agonist*) OR (aggresive*) OR (“habitat* choos*”) OR (“right* reflex*”) OR (“dispersal* abilit*”) OR (“activit* pattern*”) OR (“migrato* activit*”) OR (“reproduct* migrato*”) OR (immobilit*) OR (“territor* overlap”) OR (“free-spawn*”) OR (“egg carr*”) OR (unresponsiv*) OR (responsiv*) OR (battl*) OR (speed*) OR (“subsurfac* deposit”) OR (“ecosystem* engineer*”) OR (invasiv*) OR (“synerg* effect*”) OR (“range* exten*”) OR (“soci* interact*”) OR (“sex* interact*”) OR (intruder*) OR (repel*) OR (negoti*) OR (vigorou*) OR (compet*) OR (coal*) OR (settlement*) OR (precopulat*) OR (copulat*) OR (poleward*) OR (landward*) OR (dispersal*) OR (strik*) OR (“substrat* choic*”) OR (escap*) OR (displac*) OR (“thermal avoid*”) OR (“temperat* resist*”) OR (klinokines*) OR (orthokines*) OR (resist*) OR (thermosensitiv*) OR (“range exp*”) OR (“range contract*”) OR (distribut*) OR (migrat*) OR (beyond) OR (settle*) OR (resettlement)) | 1,703 | 15/20  (75%) | Even though the volume is lower but the comprehensiveness is not at maximum, The author decides to use search strategy #2 as the volume is not that different. |
| 4 | Population + *Exposure* + 1^st^ Outcome + 2^nd^ Outcome | TS | **POPULATION AND EXPOSURE AND 1^ST^ OUTCOME AND =** | 9,391 | 20/20 | Chosen search string: acceptable number of search hits, maximum comprehensiveness, and appropriate combination of key words responding to the project objectives and the stakeholders’ demands. |
