# Supplementary material for: What evidence exists for the impact of climate change on the physiology and behaviour of important aquaculture marine crustacean species in Asia? A systematic map protocol
Source: Environ Evid. 2022 Mar 12;11:9. doi: 10.1186/s13750-022-00263-1 (PMC11378817; doi:10.1186/s13750-022-00263-1)
Supplement: Supplementary file 3 — Additional file 3: Article for benchmarking. [file 13750_2022_263_MOESM3_ESM.docx]

**Additional File 3: Article for Benchmarking.**

| Title | Authors | Journal | Type of articles |
| --- | --- | --- | --- |
| Effects of temperature, salinity, body length, and starvation on the critical swimming speed of whiteleg shrimp, *Litopenaeus vannamei* | Yu Xiaoming, Zhang Xiumei, Duan Yan, Zhang Peidong, Miao Zhenqing | Comparative Biochemistry and Physiology A-Molecular and Integrative Physiology | Research |
| Effect of temperature on the physiology and bioenergetics of adults of the Chinese mitten crab *Eriocheir sinensis*: considerations for a species invading cooler waters | Jakubowska Magdalena and Normant Monika | Marine and Freshwater Behaviour And Physiology | Research |
| Cold-resistant changes in heartbeat of the Japanese spiny lobster | Kuramoto Tatsuya | Comparative Biochemistry and Physiology A-Molecular and Integrative Physiology | Research |
| Off-season maturation and spawning of the pacific white shrimp *Litopenaeus vannamei* in sub-tropical conditions | Kumlu Metin, Turkmen Serhat, Kumlu Mehmet, Eroldogan O. Tufan | Turkish Journal of Fisheries and Aquatic Sciences | Research |
| Critical thermal maxima and minima of *Macrobrachium rosenbergii* (Decapoda : Palaemonidae) | Fernando Dı́az Herrera, Elizabeth Sierra Uribe, L. Fernando Bückle Ramirez, Arturo Garrido Mora | Journal of Thermal Biology | Research |
| The effect of different acclimation temperatures on the prophenoloxidase system and other defence parameters in *Litopenaeus vannamei* | Pan Lu-Qing, Hu Fa-Wen, Jing Fu-Tao, Liu Hui-Jie | Fish and Shellfish Immunology | Research |
| Thermal tolerance of *Litopenaeus vannamei* (Crustacea: Penaeidae) acclimated to four temperatures | Kumlu Metin, Turkmen Serhat, Kumlu Mehmet | Journal of Thermal Biology | Research |
| Transcriptional changes revealed water acidification leads to the immune response and ovary maturation delay in the Chinese mitten crab *Eriocheir sinensis* | Luo Bi-yun, Qian Hong-li, Jiang Hu-cheng, Xiong Xin-yi, Ye Bao-qing, Liu Xue, Guo Zi-qi, Ma Ke-yi | Comparative Biochemistry and Physiology D-Genomics and Proteomics | Research |
| Effect of dissolved carbon dioxide on oxygen consumption in the Pacific white shrimp, *Litopenaeus vannamei* (Boone 1931) | Furtado Plinio S., Valenzuela Manuel A.J., Badillo Maribel A., Gaxiola Gabriela, Wasielesky Wilson, Jr. | Marine and Freshwater Behaviour and Physiology | Research |
| The effects of different types of stress on blood glucose in the giant tiger prawn *Penaeus monodon* | Hall Mike and van Ham, E. H. | Journal of the World Aquaculture Society | Research |
| Extraction of polyunsaturated fatty acids from crab ovaries *Eriocheir sinensis* by supercritical carbon dioxide | Zhu Beiwei, Zhou Dayong, Yang Jingfeng, Liu ZhaoFang | Journal of Biotechnology | Research |
| The effects of high concentration of carbon dioxide on performance and tissue histology of shrimp *Litopenaeus vannamei* | Casillas-Hernandez Ramon, Arevalo-Sainz Karla Janeth, Gonzalez-Galaviz Jose Reyes, Rodriguez-Jaramillo Maria del Carmen, Borquez-Lopez Rafael Apolinar, Gil-Nunez Juan Carlos, Flores-Perez Maria Belem, Lares-Villa Fernando, Ibarra-Gamez Jose Cuauhtemoc, Molina Barrios Ramon M. | Aquaculture Research | Research |
| Growth of postlarval *Macrobrachium rosenbergii* at two temperatures | Arana-Magallon E.C. and Ortega-Salas Armando Adolfo | North American Journal of Aquaculture | Research |
| Impact of elevated temperature on physiological energetics of *Penaeus monodon* post larvae: A mesocosm study | Nandy Tanmoy, Baag Sritama, Mandal Sumit | Journal of Thermal Biology | Research |
| Thermal preference, tolerance and oxygen consumption of adult white shrimp *Litopenaeus vannamei* (Boone) exposed to different acclimation temperatures | Gonzalez Ricardo A., Diaz Fernando, Licea Alexei, Denisse Re Ana, Noemi Sanchez L., Garcia-Esquivel Zaul | Journal of Thermal Biology | Research |
| Physiological tolerance of the early life history stages of fresh water prawn (*Macrobrachium rosenbergii* De Man, 1879) to environmental stress | John Jojy, Siva Vinu S., Kumar Amit | Indian Journal of Geo-Marine Sciences | Research |
| Effects of temperature on complexes I and II mediated respiration, ROS generation and oxidative stress status in isolated gill mitochondria of the mud crab *Scylla serrata* | Paital Biswaranjan and Chainy Gagan B.N. | Journal of Thermal Biology | Research |
| The influence of temperatures ranging from 25 to 36 degrees C on developmental rates, morphometrics and survival of freshwater prawn (*Macrobrachium rosenbergii*) embryos | Saydmohammed Manush, Pal Asim Kumar, Das T., Mukherjee S.C. | Aquaculture | Research |
| Effects of temperature on feeding and activity in the crab *Scylla-Serrata* | Hill, Bryony J. | Marine Biology | Research |
| Effect of environmental embryonic temperature on larval development of *Macrobrachium-rosenbergii* (deman) | Garcia Diaz, Grettel | Journal of Experimental Marine Biology and Ecology | Research |
